# Adult abuse and poor prognosis in Taiwan, 2000–2015: a cohort study

**DOI:** 10.1186/s12889-022-14663-y

**Published:** 2022-12-06

**Authors:** Pi-Ching Yu, Ya-Hsuan Chiang, Shi-Hao Huang, Ren-Jei Chung, Iau-Jin Lin, Bing-Long Wang, Chia-Peng Yu, Yu-Ching Chou, Chien-An Sun, Shih-En Tang, Yao-Ching Huang, Wu-Chien Chien, Chun-Hsien Chiang

**Affiliations:** 1 Cardiovascular Intensive Care Unit, Department of Critical Care Medicine, Far-Eastern Memorial Hospital, 10602 New Taipei City, Taiwan; 2 Graduate Institute of Medicine, National Defense Medical Center, 11490 Taipei, Taiwan; 3 Cardiovascular Medicine, Far Eastern Memorial Hospital, 10602 New Taipei City, Taiwan; 4 Department of Medical Research, Tri-Service General Hospital, 11490 Taipei, Taiwan; 5 Department of Chemical Engineering and Biotechnology, National Taipei University of Technology (Taipei Tech), 10608 Taipei, Taiwan; 6 School of Public Health, National Defense Medical Center, 11490 Taipei, Taiwan; 7 Department of Public Health, College of Medicine, Fu-Jen Catholic University, 242062 New Taipei City, Taiwan; 8 Big Data Center, College of Medicine, Fu-Jen Catholic University, 242062 New Taipei City, Taiwan; 9 Department of Internal Medicine, Division of Pulmonary and Critical Care Medicine, Tri‑Service General Hospital, National Defense Medical Center, 11490 Taipei, Taiwan; 10 Institute of Aerospace and Undersea Medicine, National Defense Medical Center, 11490 Taipei, Taiwan; 11 Graduate Institute of Life Sciences, National Defense Medical Center, 11490 Taipei, Taiwan; 12 Taiwanese Injury Prevention and Safety Promotion Association (TIPSPA), 11490 Taipei, Taiwan; 13 Department of Cardiovascular Medicine, Far-Eastern Memorial Hospital, New Taipei City, Taiwan

**Keywords:** Adult maltreatment, Psychotic disorders, Schizophrenic disorders, Suicide, Self-inflicted injury, Violent injury

## Abstract

**Background:**

To investigate the risk of poor prognosis regarding schizophrenic disorders, psychotic disorders, suicide, self-inflicted injury, and mortality after adult violence from 2000 to 2015 in Taiwan.

**Methods:**

This study used data from National Health Insurance Research Database (NHIRD) on outpatient, emergency, and inpatient visits for two million people enrolled in the National Health Insurance (NHI) from 2000 to 2015. The case study defined ICD-9 diagnosis code N code 995.8 (abused adult) or E code E960-E969 (homicide and intentional injury of another). It analyzed first-time violence in adults aged 18–64 years (study group). 1:4 ratio was matched with injury and non-violent patients (control group). The paired variables were sex, age (± 1 year), pre-exposure to the Charlson comorbidity index, and year of medical treatment. Statistical analysis was conducted using SAS 9.4 and Cox regression for data analysis.

**Results:**

In total, 8,726 individuals experienced violence (case group) while34,904 did not experienced violence (control group) over 15 years. The prevalence of poor prognosis among victims of violence was 25.4/10^4^, 31.3/10^4^, 10.5/10,^4^ and 104.6/10^4^ for schizophrenic disorders, psychotic disorders, suicide or self-inflicted injury and mortality, respectively. Among adults, the risks of suicide or self-inflicted injury, schizophrenic disorders, psychotic disorders, and mortality after exposure to violence (average 9 years) were 6.87-, 5.63-, 4.10-, and 2.50-times (*p* < 0.01), respectively, compared with those without violence. Among males, the risks were 5.66-, 3.85-, 3.59- and 2.51-times higher, respectively, than those without violence (*p* < 0.01), and they were 21.93-, 5.57-, 4.60- and 2.46-times higher than those without violence (*p* < 0.01) among females.

**Conclusion:**

The risk of poor prognosis regarding schizophrenic disorders, psychotic disorders, suicide, or self-inflicted injury and mortality after adult violence was higher than in those who have not experienced a violent injury. Adults at the highest risk for violent suicide or self-inflicted injuries due to exposure to violent injuries —males were at risk for schizophrenia and females were at risk for suicide or self-inflicted injuries. Therefore, it is necessary for social workers and medical personnel to pay attention to the psychological status of victims of violence.

## Introduction

 Abuse is the mishandling or use of something, usually for unfair or improper advantage [1]. Abuse can take many methods, such as physical abuse or injury, rape, assault, unfair methods, crime, or other types of assault [2]. Physical and emotional abuse is harmful and can lead to mental illness [3]. It can be associated with verbal and non-verbal abuse and can humiliate, intimidate and isolate a person's mind [4]. Using physical injury, abuse, damage or destruction as a source of violence [4]. Violence can be physical, psychological, social, financial, or sexual assault [3, 4]. Violence can be intermittent, occasional, or chronic [5]. The emotional impact of adult abuse leading to isolation, fear, and mistrust can have lifelong adverse outcomes, including poor psychosomatic and behavioral health and increased risk of adverse outcomes [6]. Dating violence, domestic violence, human trafficking, child abuse and neglect, sexual assault and elder abuse are relationship violence [7]. Abuse can happen at any time and to anyone and has severe, lasting negative emotional, mental, and physical effects on the victim [8]. Violent injury and suicide are closely related major public health issues [9].

World Health Organization (WHO) estimates that it is estimated that hundreds of thousands of people worldwide die by suicide every year [10]. Complex combination of demographic characteristics, social risk factors, mental illness and substance abuse, and multilevel causality lead to suicide [11–18]. Previous research has found strong associations between adult abuse and suicide, mental health problems, substance abuse, social isolation, trauma, and violence [19]. Murder-suicide cases most likely in context of abuse, intimate partner violence (IPV) survivors twice as likely to make multiple suicide attempts [20].

A better understanding of the physical and mental health effects on adults who experience violence and suicide can help lift public health efforts to the next level. Longitudinal observational studies on the relationship between adult abuse and poor prognosis (schizophrenic disorders, psychotic disorders, suicide, or self-inflicted injuries and mortality) are limited. Therefore, we hypothesized that adults exposed to violence risk poor prognoses (schizophrenic disorders, psychotic disorders, suicide, or self-inflicted injury and mortality). We used NHIRD to track whether adults experiencing violence were at risk of poor prognosis (schizophrenic disorders, psychotic disorders, suicide, or self-inflicted injury and mortality) from 2000 to 2015 through long-term follow-up in Taiwan.

## Method

### Data sources

 According to statistics from the Gender Equality Committee of the Executive Yuan, the actual coverage rate of NHI increased from 99.29% to 1998 to 99.93% in 2020. This study used NHIRD's 2000 NHI Parents Cohort (Longitudinal Health Insurance Research Database, LHID2000) from January 1, 2000 to December 2015 to December 31, 2015 as the research data source; outpatient and inpatient data were followed up for 16 years. In this study, data from 2000 were used for data cleaning, and non-new cases were excluded. According to the International Classification of Diseases, Ninth Revision, Clinical Revision (ICD-9 CM) N-code995.8, and Exogenous Classification Code E-code: E960 - E969, adults subjected to violence in this study were 18–64 years old.

The control group was established by case-control matching method with a matching ratio of 1:4. Matching criteria included sex, age (±1 year), time of primary violence occurrence (month and year) (same year and month of treatment in the control group), and accumulated to the Charlson Comorbidity Index (CCI) prior to first exposure). CCI before violence was adjusted. CCIs with scores above 10 were classified at the same level according to the actual situation of the data to find enough control casess. The other were matched with the original CCI scores according to the data. The paired data were statistically analyzed. Deyo revised the CCI in 1992 [21]. Adult abuse ICD9 codes 800-989, 995.80-995.85. Personally identifiable information is not included in the analyzed data. Tri-Service General Hospital Ethical Review Board of the National Defense Medical Center (TSGHIRB number: C202105014) approved the study and waived the requirement for written informed consent. The source of funding for this project is the scientific research project of the General Hospital of the National Defense Medical College (TSGH-B-111018).

### Study design

A retrospective matched cohort design was used in this study from January 1, 2000, to December 31, 2015. ICD-9-CM code 995.80 is for the adult abuse Index. Adult abuse cohort included victims over 18 years old (n = 8,726). In addition, a comparison cohort of 34,904 matched sex, age, CCI, and index date (1:4) without any experience of adult abuse was included. Poor prognosis includes schizophrenic and psychotic disorders, suicide, self-inflicted injury, and mortality. Schizophrenia is a serious mental illness in which the sufferer interprets the status quo abnormally. Schizophrenia causes hallucinations, delusions, and extremely confused thinking, which impair daily functioning and can cause disability. The two main symptoms of schizophrenia are delusions and hallucinations. Suicide is death by injuring yourself. A suicide attempt occurs when someone injures themselves with any intent to end their own life, meaning they did not die as a result of their suicide act. Self-inflicted injury occurs when a person intentionally harms their own life. Self-injury is also known as self-abuse, intentional self-injury, suicidal-like behavior, or non-suicidal self-injury. Victims with adult abuse experience or poor prognosis before the index date (persons who first presented with abuse during the study period) or 2000 were excluded (individuals who could have presented with abuse at some point during the study period before the index act was excluded).

Covariates included sex, age, geographic place of residence (Northern region, Central region, Southern region or Eastern region), level of urbanization (level 1-4), level of hospital (medical center, regional hospital, district hospital) and type of insurance cost ​​​(New Taiwan Dollars [NTD]; <18,000, 18,000–34,999, ≥ 35,000).

The level of urbanization is based on population density (person/km^2^), the proportion of the population with a college education or above (%), the proportion of the population over the age of 65 (%), the percentage of the population classified as agricultural workers (%), and the number of physicians per 100,000 people in the county [22].

This study applied the CCI score of 17 relevant comorbidity categories (based on ICD-9-CM codes) [15]. CCI scores ranged from 0 to 37 and indicate comorbidities from minor health problems.

**Fig. 1:**
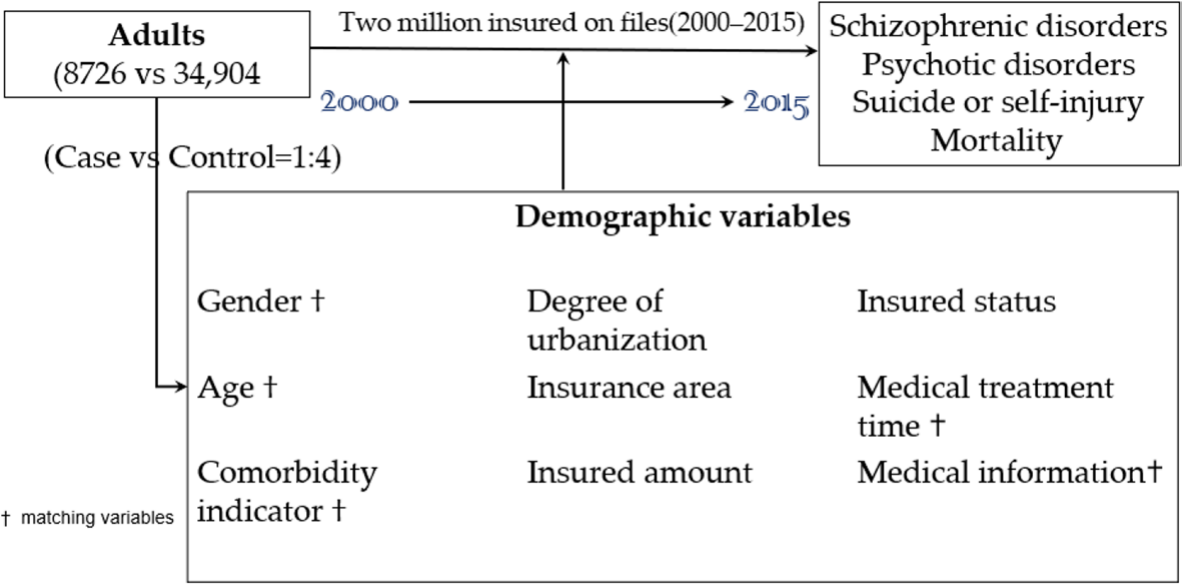
The flowchart overview of the study design

The study follow-up period was January 1, 2000, until the onset of poor prognosis (schizophrenic disorders, psychotic disorders, suicide, or self-inflicted injury and mortality) until 2015. A flowchart of the study design is presented in Fig. 1.

### Statistical analysis

SAS 9.4 for Windows (SAS Institute, Cary, NC, USA) statistical software was used for analysis, and the generalized estimating equation (GEE) method was used to test the chi-square test, logistic regression, hierarchical Cox regression, paired and descriptive data of the two groups.

## Results

### Characteristics of study

A case group of 8,726 abused adults and a matched control group of 34,904 non-abuse adults were included in the study. The average age of violently abused adults was 36.9±12.0 years old, and males accounted for 67.1%. Insurance premiums, CCI, level of urbanization, and level of care were different between the two groups (Table 1).

**Table 1:** Data of the adult violence victim group and the medical treatment control group

Demographic Variables		Case Group (*n* = 8726)		Control Group (*n* = 34,904)		*p-value*
		***n***	***%***	***n***	***%***	
Sex	Women	2873	32.9	11,492	32.9	> 0.999
	Men	5853	**67.1**	23,412	67.1	
Age	Mean (SD)	36.9	(12.4)	36.9	(12.2)	0.9279
	Median (Min-Max)	36	(18–64)	36	(18–64)	
Degree of Urbanization	Highly urbanized Town	1758	**20.1**	10,157	29.1	< 0.001
	Moderately urbanized town	2513	28.8	11,255	32.2	
	New town	1934	22.2	6244	17.9	
	General townships	1340	15.4	4416	12.7	
	Aging towns + Remote towns + Outlying islands	716	8.2	1502	4.3	
	Agricultural township	430	4.9	955	2.7	
	Missing value	35	0.4	375	1.1	
Geographical Area	Taipei	2136	24.5	12,768	36.6	< 0.001
	North District	1124	12.9	5081	14.6	
	Central District	2408	27.6	6238	17.9	
	South area	1034	11.8	4725	13.5	
	Kaohsiung and Pingtung	1737	**19.9**	5013	14.4	
	Eastern District	260	3.0	741	2.1	
	Missing value	27	0.3	338	1.0	
Insured Amount	0-16499 yuan	2870	32.9	5894	16.9	< 0.001
	16,500–20,999 yuan	2464	28.2	9434	27	
	21,000–30,299 yuan	2006	23.0	8238	23.6	
	≧ 30,300 yuan	1386	15.9	11,338	32.5	
Personal or Health Insurance	Public insurance	233	2.7	1997	5.7	< 0.001
Dependent Occupation	Labor protection	4542	52.1	23,870	68.4	
	Farmer	841	9.6	2710	7.8	
	Member of Water Conservancy and Fisheries Association	317	3.6	866	2.5	
	Low-income Households	208	2.4	263	0.8	
	Community insured Population	2557	29.3	4844	13.9	
	Other + missing Values	28	0.3	354	1.0	

by *GEE*, correlation matrix: unstructured

### Mental illness risk and poor outcomes according to adult abuse exposure

Adult victims of violence are at significantly higher risk of developing schizophrenia, psychosis, suicide, self-harm, and death than victims who have not experienced such violence. Among them, the most significant differences in risk were suicide and self-harm (HR=6.87), schizophrenia (HR=5.63), mental disorders (HR=4.10) and death risk (HR=2.50) (Table 2).

**Table 2:** Risk of adverse outcomes for victims of violence compared to those who did not experience violence (adults, overall)

Poor Prognosis	Case Group (*n* = 8,726)	Control Group (*n* = 34,904)	HRs (95% CI)	*p*-value
	**Incidence (1/10**^**4**^**)**	**Incidence ** **(1/10**^**4**^**)**		
Psychiatric comorbidity				
Schizophrenic disorders	25.4	4.9	5.63(4.44–7.14)	< 0.0001
Psychotic disorders	31.3	8.4	4.10 (3.36–4.99)	< 0.0001
Suicide or self-inflicted injury	10.5	1.5	6.87(4.64–10.17)	< 0.0001
Mortality	104.6	41.1	2.50 (2.26–2.76)	< 0.0001

The stratified Cox regression analysis, corrected CCI, insurance amount, insurance status, sex, age, and CCI before pairing were corrected at the time of pairing. The reference group was non-violent. The incidence rate (unit) was 1/10^4^. HRs were based on adjusted rather than crude analyses

Adult male victims of violence are at significantly higher risk of schizophrenic disorders, psychotic disorders, suicide or self-inflicted injury, and mortality than the victims who did not experience violence. The risk differences of schizophrenia (HR=5.66), mental disorders (HR=3.85), suicide or self-injury (HR=3.59) and death risk (HR=2.51) were the most significant for poor prognosis (Table 3).

**Table 3:** Risk of adverse outcomes for victims of violence compared to those who did not experience violence (male adults)

Poor prognosis	Case Group (*n* = 5,853)			Control Group (*n* = 23,412)			HRs (95% CI)	*p*-value
	***n***	**%**	**Incidence ** **(1/10**^**4**^**)**	***n***	**%**	**Incidence (1/10**^**4**^**)**		
Psychiatric omorbidity								
Schizophrenic disorders	118	2.0	24.6	91	0.4	4.7	5.66(4.22–7.59)	< 0.0001
Psychotic disorders	138	2.4	28.7	162	0.7	8.3	3.85 (3.01–4.91)	< 0.0001
Suicide or self-inflicted injury	32	0.5	6.6	34	0.1	1.7	3.59(2.18–5.90)	< 0.0001
Mortality	603	10.3	124.3	957	4.1	49.2	2.51(2.25–2.79)	< 0.0001

The stratified Cox regression analysis, corrected CCI, insurance amount, insurance status, sex, age, and CCI before pairing were corrected at the time of pairing. The reference group was non-violent. The incidence rate (unit) was 1/10^4^. HRs were based on adjusted analyses instead of crude analyses

Females adult victims of violence had a significantly higher risk of suicide or self-inflicted injury (HR = 21.93), schizophrenic disorders (HR = 5.57), psychotic disorders (HR = 4.60), and the risk of mortality (HR = 2.46). (Table 4).

**Table 4:** Risk of adverse outcomes for victims of violence compared to those who did not experience violence (female adults)

Poor prognosis	Case Group (*n* = 2,873)			Control Group (*n* = 11,492)			*HRs (95% CI)*	*p-value*
	**n**	**%**	**Incidence (1/10** ^**4**^ **)**	**n**	**%**	**Incidence (1/10** ^**4**^ **)**		
Psychiatric comorbidity								
Schizophrenic disorders	58	2.0	27.3	48	0.4	5.5	5.57(3.71–8.35)	< 0.0001
Psychotic disorders	79	2.7	37.2	73	0.6	8.4	4.60(3.30–6.40)	< 0.0001
Suicide or self-inflicted injury	41	1.4	19.3	7	0.1	0.8	21.93(9.81–49.02)	< 0.0001
Mortality	129	4.5	60.1	200	1.7	23.1	2.46(1.94–3.12)	< 0.0001

The stratified Cox regression analysis, corrected CCI, insurance amount, insurance status, sex, age, and CCI before pairing were corrected at the time of pairing. The reference group was non-violent. The incidence rate (unit) was 1/10^4^. HRs were based on adjusted analyses instead of crude analyses

## Discussion

The findings showed that adults who experienced violence had a significantly higher risk of developing schizophrenia, psychosis, suicide or self-harm, and death) than adults who did not experience violence. The risk of poor prognosis (adults overall) was suicide or self-inflicted injury (HR = 6.87), schizophrenic disorders (HR = 5.63), psychotic disorders (HR = 4.10), and risk of mortality (HR = 2.50). The risk of poor prognosis (male adults) was schizophrenia (HR = 5.66), psychotic disorders (HR = 3.85), suicide or self-inflicted injury (HR = 3.59), and risk of mortality (HR = 2.51). The risk of poor prognosis (female adults) was suicide or self-inflicted injury (HR = 21.93), schizophrenic disorders (HR = 5.57), psychotic disorders (HR = 4.60), and risk of mortality (HR = 2.46). Adults following violent injury are at highest risk for suicide or self-inflicted. Male adults suffer from violence were at the highest risk of developing schizophrenia. Further, female adults suffer from violence were at the highest risk of suicide or self-inflicted injuries. Suicidal behavior is strongly associated with experiencing conflict, disaster, violence, abuse or loss, and feelings of isolation [23]. In addition, refugees and immigrants experience high rates of suicide among discriminated populations [24]. Men and women may develop schizophrenia at the same rate, but men tend to develop schizophrenia earlier [25]. On average, males are diagnosed with schizophrenia in their late teens to early 20s, while females are diagnosed with schizophrenia in their late 20s to early 30s [26]. Females are nine times more likely to experience IPV than males [27].  Females are twice to suffer from Post-Traumatic Stress Disorder (PTSD) [28]. IPV can lead to PTSD, which is also a risk factor for suicide [29]. Suicide or self-harm are unhealthy strategies that women may use to cope with abuse [30], which is consistent with our study.

Mental illnesses, particularly PTSD, depression, anxiety, and alcohol use disorders (AUD), are well-known risk factors for suicide and self-harm [31]. PTSD is a known risk factor for self-harm and suicide [31, 32]. 10% of women suffer from PTSD at some point in their lives, compared to 4% of men. PTSD study shows women are twice as likely as men to suffer from PTSD [33]. Young women are more likely than men to experience high-impact trauma [34]. When type II trauma is involved, it disrupts neurobiological development and personality [33, 34]. 

 Estimates published by WHO indicate that approximately one-third (30%) of women worldwide have experienced physical or sexual intimate partner violence or non-partner sexual violence during their lifetime [35]. Violence leads to unhealthy coping strategies that can ultimately destroy self-worth, lead to PTSD and depression, and put women at greater risk of suicide [36]. Our study found that adults exposed to violence were at the most significant risk of suicide or self-inflicted injuries. Women were at the highest risk of suicide and self-inflicted injury due to violence.

Schizophrenia that affects a person’s thinking, behavior, emotional expression, perception of reality, and relationships with others [37]. Although schizophrenia is not as common as other major mental illnesses, it can be the most chronic and disabling [38]. Individuals who experienced emotional abuse in early life were 3.5 times more likely to experience schizophrenia in adulthood [39]. The more severe the abuse, the more severe the schizophrenia-like experiences in adults [40]. Exposure to violence, traumatic brain injury, or substance intoxication can act as a single trigger to increase risk of short-term violence in schizophrenia and controls [41, 42]. Our study found that male adults exposed to violence were at the highest risk of developing schizophrenic disorders.

In today’s society, psychotic disorders and violent abuse are often inextricably linked, creating a harsh stigma for patients and sometimes an uncomfortable environment for psychiatrists [43]. Dealing with violent injuries in victims have become a growing concern in the psychiatric practice. Numerous patients of assault present to emergency departments, and psychiatrists are often called upon to assess and treat such violently injured patients [44]. Victims of violence have a much higher risk of having mental health disorders than the general population [45–50]. Our study found that adults exposed to violence risk psychotic disorders.

Every year, more than 1.6 million people die from violence worldwide [51]. Victims of violence are associated with excess mortality and risk of suicide [52]. It is essential to systematically screen for violence victimization in clinical settings to identify at-risk individuals [53]. Similarly, in clinical work, patients with victimization experiences should be screened for substance abuse and should be treated comprehensively [54]. Our study found that adults exposed to violence were at risk of mortality.

Several limitations in the study. First, similar to previous studies that used the NHI Mental Illness Research Database [55], we were unable to assess genetic psychosocial or environmental factors, severity, or psychological assessment in individuals with mental disorders because data were not recorded in NHIRD. Second, the study was conducted on adults who had experienced violence and sought medical care. so investigators only examine those who have experienced more severe violence, whose incidence may be underestimated. Third, this study examined medical violence cases, which may minimize the harm caused by violence. In particular, domestic violence cases and violence without obvious trauma or other unexplained psychological abuse events: Victims that did not seek medical treatment.

## Conclusion

The risk of poor prognosis (schizophrenic disorders, psychotic disorders, suicide, or self-inflicted injury and mortality) among adults subjected to violence is significantly higher than those who were not been subjected to violence. Adults who have experienced violence are at the highest risk for suicide or self-harm. Males who experience violence are at highest risk for schizophrenia. Females who experience violence are at the highest risk of suicide or self-harm. Therefore, in addition to avoiding violent incidents, medical and social welfare personnel should also be concerned with the mental health and risks of schizophrenia, mental disorders, suicide, self-harm, and death in violent adults. 

Future studies should investigate changes in poor prognosis (schizophrenic disorders, psychotic disorders, suicide, or self-inflicted injury and mortality) over the observation period from 2016 to 2022.

## Data Availability

Data are available from the National Health Insurance Research Database (NHIRD) published by the Taiwan National Health Insurance (NHI) Administration. Due to the legal restrictions imposed by the government of Taiwan concerning the “Personal Information Protection Act,” data cannot be made publicly available. Requests for data can be sent as formal proposals to the NHIRD (https://dep.mohw.gov.tw/DOS/lp-2506-113.html).
